# Double non-contiguous fractures in a patient with spondylo-epiphyseal dysplasia with spinal ankylosis treated with open and percutaneous spinal fixation technique: a case report

**DOI:** 10.1186/s13104-018-3227-7

**Published:** 2018-02-07

**Authors:** Takahiro Ushijima, Kenichi Kawaguchi, Tadashi Matsumoto, Masaki Takagi, Tatsuro Kondoh, Gen Nishimura, Aritoshi Iida, Shiro Ikegawa, Nobuhiko Haga, Go Kato

**Affiliations:** 1 Department of Spine Surgery, Saga Medical Centre, Koseikan, 400 Nakabaru Kase-Machi, Saga, 840-8571 Japan; 2 Department of Trauma Centre, Saga Medical Centre, Koseikan, 400 Nakabaru Kase-Machi, Saga, 840-8571 Japan; 3 Department of Orthopedic Surgery, Kyushu University Graduate School of Medical Sciences, 3-1-1 Maidashi Higashi-Ku, Fukuoka, 812-8582 Japan; 4 Division of Developmental Disability, Misakaenosono Mutsumi Developmental Medical and Welfare Center, 570-1 Konagaichomaki, Isahaya, 859-0164 Japan; 5 Department of Endocrinology and Metabolism, Tokyo Metropolitan Children’s Medical Center, 2-8-29 Musashisdai Fuchu, Tokyo, 183-8561 Japan; 6 Department of Radiology, Tokyo Metropolitan Children’s Medical Center, 2-8-29 Musashisdai Fuchu, Tokyo, 183-8561 Japan; 7 Laboratory of Bone and Joint Diseases, RIKEN Center for Integrative Medical Sciences, 4-6-1 Shiroganedai Minato-Ku, Tokyo, 108-8639 Japan; 8 Department of Rehabilitation Medicine, Graduate School of Medicine, The University of Tokyo, 7-3-1 Hongo Bunkyo-Ku, Tokyo, 113-8655 Japan

**Keywords:** Trauma, Spine, Spondylo-epiphyseal dysplasia, Ankylosing spine, Spinal fracture

## Abstract

**Background:**

Patients with ankylosing spines are susceptible to developing spinal fractures even with minor trauma and can develop early or late neurological injuries. These fractures require early and aggressive surgical management to enable spinal stability and/or neural decompression. Being highly unstable by nature, they require relatively long segment instrumentation and fusion, which can increase paravertebral soft tissue damage and perioperative bleeding. The purpose of this report is to describe a rare case of traumatic double fractures at the cervico-thoracic and thoraco-lumbar transition zones in ankylosing spine with spondylo-epiphyseal dysplasia (SED) of unknown cause, which were successfully treated with a combined open and percutaneous spinal fusion procedure.

**Case presentation:**

A 46-year-old woman who was diagnosed with non-contiguous fractures in cervico-thoracic and thoraco-lumbar junction zones among multiple injuries sustained in a traffic accident was treated with hybrid techniques for posterior instrumentation with an open approach using a computed tomography (CT)-based navigation system and percutaneous pedicle-screwing method. She regained mobility to pre-admission levels and started walking on crutches 3 months postoperatively. Genetic testing for the cause of SED revealed no mutation in the COL2A1 or TRPVR4 genes. The union of fractured spine was confirmed on CT scan 1 year postoperatively.

**Conclusion:**

This is the first report of double spinal fractures in an ankylosing spine with genetically undetermined spondyloepiphyseal dysplasia. A long-segment posterior instrumentation procedure incorporating the invasive treatment of spinal fractures in ankylosing spondylitis or diffuse idiopathic hyperostosis was effective.

## Background

Ankylosing spondylitis (AS) or diffuse idiopathic skeletal hyperostosis (DISH) commonly accompanies ankylosing spines; spinal fractures frequently occur with minor trauma [1, 2] and are extremely unstable because of the long lever arms of the fused spinal column and several complications, including common early and late neurological symptoms [2]. Therefore, early surgery for neural decompression and spinal stability is recommended [3, 4]. A case with double fractures of an ankylosing spine with genetically undetermined spondyloepiphyseal dysplasia (SED) and spinal ankylosis was successfully treated with hybrid open and percutaneous spinal fusion.

## Case presentation

**Fig. 1:**
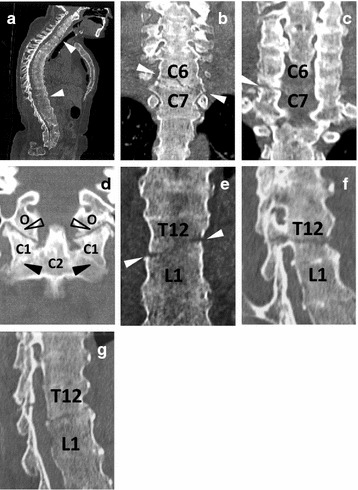
**a** Sagittal image of the whole spine demonstrates an ankylosing spine and unclear intervertebral discs. **b**, **c** Spinal fracture at the cervico-thoracic junction zone. Coronal sections show a fracture line going from the right C6 vertebral body to the left C6/7 intervertebral space (arrow heads) (**b**) and a gap at the left C6/7 facet (**c**). **d** Note that all other cervical facets including atlanto-axial joints (filled arrow heads) are ankylosed, and incongruity of atlanto-occipital joints (open arrow heads) are not observed. **e**–**g** A chance fracture at the thoraco-lumbar junction zone. This coronal section shows a fracture line that goes from the left T12 vertebral body to the right T12/L1 intervertebral space (arrows) (**e**). Left and right parasagittal section (**f**, **g**) demonstrates fracture lines through the pedicle (**f**) and lamina (**g**)

A 46-year-old woman involved in a traffic accident was brought to our hospital. On arrival at the emergency room, her vital signs were stable and physical examination revealed no neurological deficit. Whole body computed tomography (CT) confirmed double spinal fractures at the cervicothoracic (C-T) and thoracolumbar (T-L) junctional zones in an ankylosing spine, with atlas to coccyx fusion, as well as traumatic hemopneumothorax and multiple rib fractures. A right thoracic curve with a Cobb angle of 15°, a sagittal kyphotic thoracic curve with a Cobb angle of 73°, sacral anteversion, and coccygeal retroversion were observed **(**Fig. 1**)**. Magnetic resonance imaging of the spine revealed a mild dural sac indentation at the T-L junctional zone, but without spinal cord compression.

**Fig. 2:**
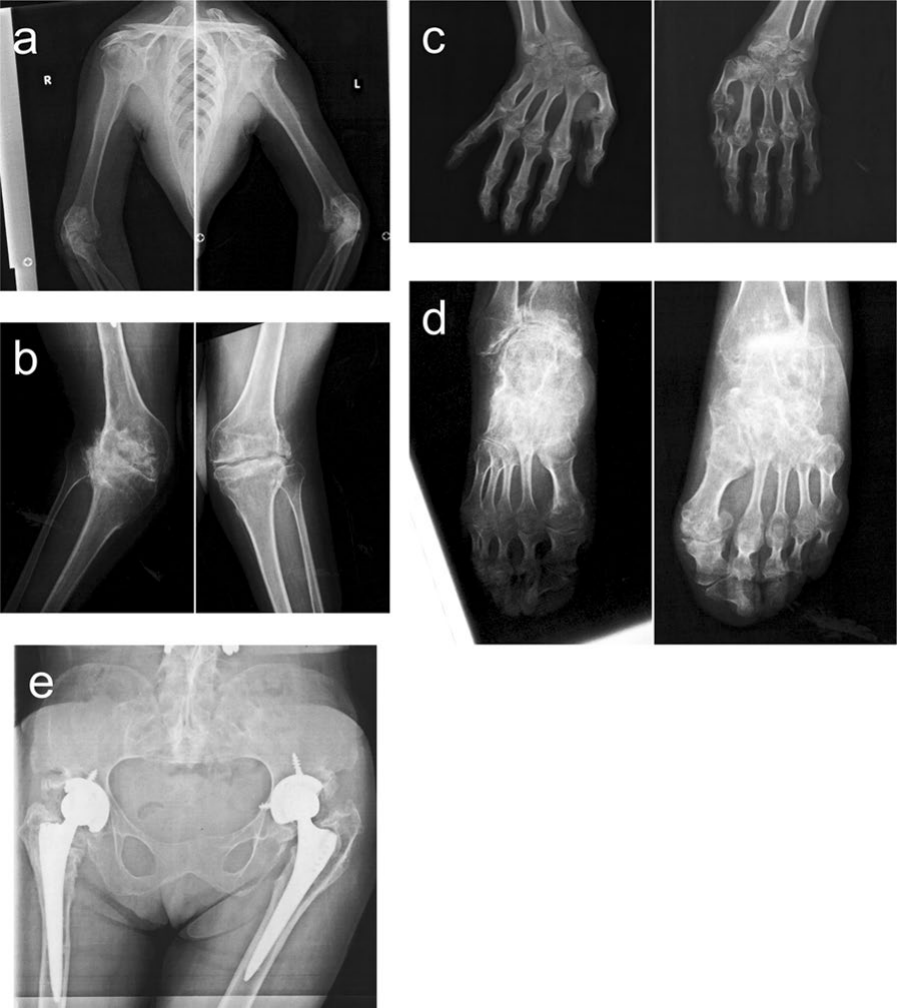
Almost all of the large (**a**, **b**) and small joints of the extremities (**c**, **d**), sacroiliac joints, and pubic symphysis **e** show marked osteoarthritic changes and/or ankylosis. Bilateral joint replacements had been performed for hip joint osteoarthritis

**Fig. 3:**
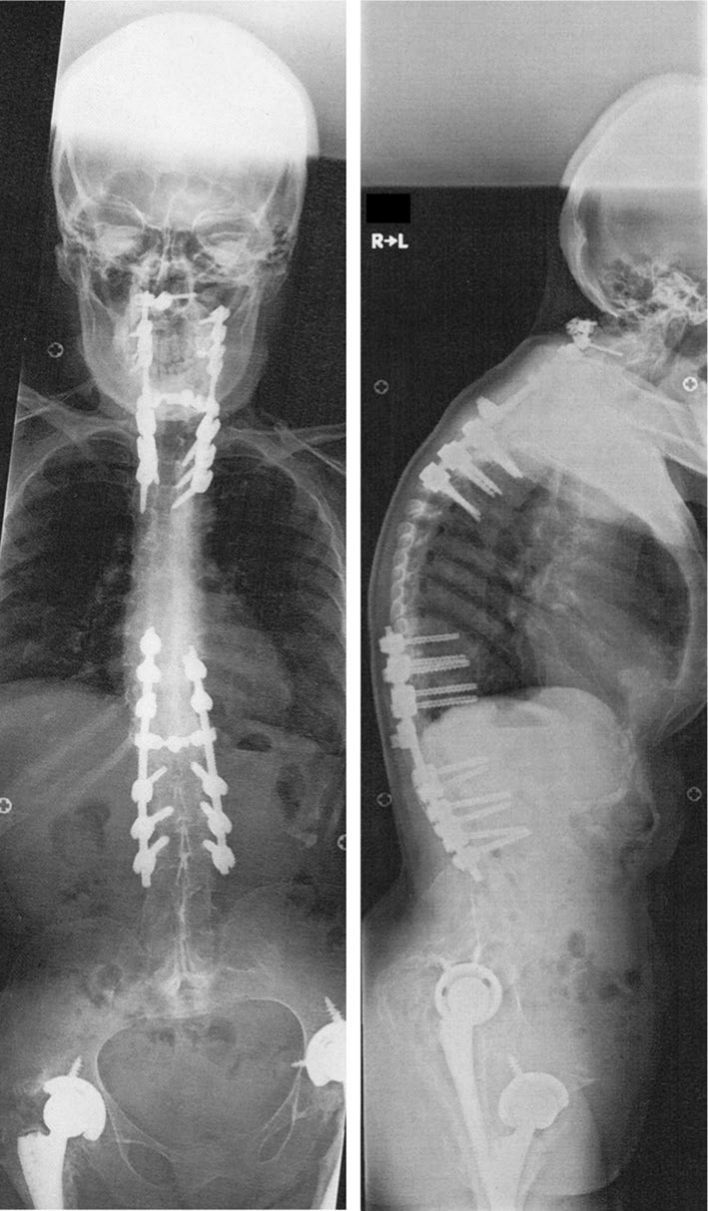
The anteroposterior (left) and lateral (right) roentgenograms of spinal fusion of both fractures at the cervico-thoracic and thoraco-lumbar junctions without bone grafting

In infancy, the patient had progressive multiple joint contractures indicating arthrogryposis multiplex congenital, which was not genetically confirmed. Height and weight on admission were 130 cm and 40 kg, respectively. No visual or acoustic deficits were evident. Whole body roentgenograms revealed marked osteoarthritic changes in almost all joints and ankyloses in the knees and shoulders. She had no medical history of fractures and underwent left and right hip joint replacement surgeries at 32 and 39 years of age, respectively **(**Fig. 2**)**. Respiratory distress from traumatic hemopneumothorax resolved within 8 days of hospitalization, and the patient subsequently underwent surgical posterior spinal fusion of both spinal fractures without bone grafting **(**Fig. 3**)**.

The patient underwent tracheal intubation in the neutral cervical spine position using a monitor-integrated video laryngoscopy. She was then placed in the prone position taking care not to displace floating thoracic spine fragments. Spinal alignment in the prone position was checked using fluoroscopy and CT; a mattress was inserted between the body and four-post spine frame to equalize the space created by the thoracic spine kyphotic deformity, and C2–T4 and T9–L3 posterior fusion was performed. A C2 lamina screw (left side), pedicle screw (right side), cervical lateral mass screws (C3–C6), and thoracic pedicle screws (T2–T4) were inserted via an open approach. A C-arm three-dimensional navigation system (Brainlab Spinal Navigation, Munich, Germany) was used for placing C2 and thoracic pedicle screws; lateral mass screws were placed using a lateral fluoroscope. Contoured rods from C2 to T4 and a cross-link at the T1 level were placed. Pedicle screws were percutaneously placed from L1 to L3 using biplanar fluoroscopy.

**Fig. 4:**
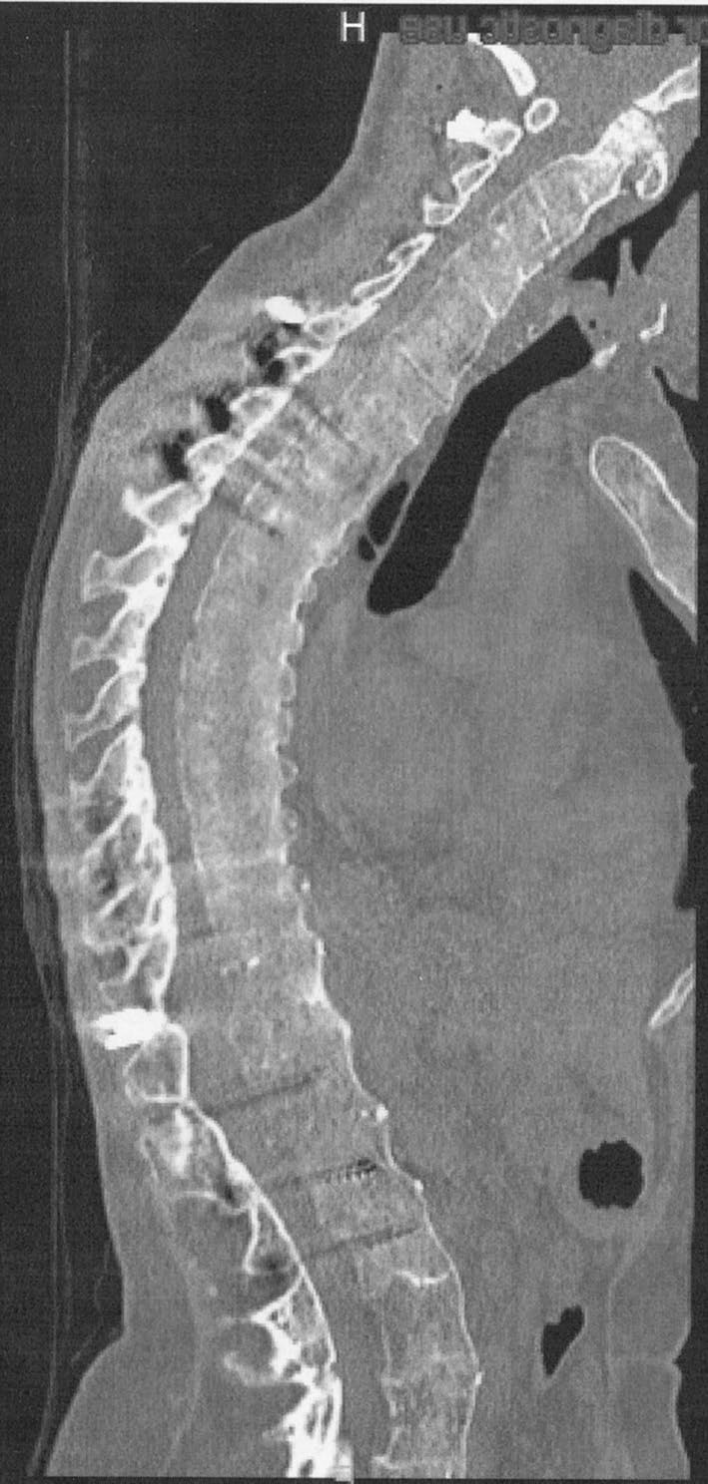
Sagittal computed tomography image of the whole spine 1 year postoperatively

Although identifying pedicle contours of T9–T11 was difficult because of spinal osteoporosis, screw placement was safely performed without a navigation system via the open approach because the diameters of the pedicles at these levels were large enough (> 6.0 mm on the preoperative CT scan). Pedicle screws were not placed along the left side of T9 because the pedicle was sclerosed and a rib fracture dislocation occurred while preparing the pilot hole. The 5.5-mm contoured rods were inserted through polyaxial heads of pedicle screws at the open thoracic incision and caudally passed subcutaneously through polyaxial heads of lumbar pedicle screws. Cross-links were placed between T12 and L1. Decompressive laminectomy or laminotomy was not performed because of the absence of cord compression. Surgical duration was 8 h, with approximately 1100 mL blood loss. The patient recovered well without postoperative complications and was discharged for rehabilitation 2 months postoperatively. She could walk and resume work, respectively, at 3 and 6 months postoperatively. One-year follow-up CT revealed a bony fusion of C-T and T-L fractures **(**Fig. 4**)**. We suspected SED based on her medical history and whole body roentgenograms, but did not find *COL2A1* and *TRPV4* mutations; further genetic testing was not performed because no candidate gene for this phenotype was identified.

## Discussion and conclusions

We report double noncontiguous spinal fractures in a patient with SED having spinal ankylosis. No similar cases have been reported, which necessitated adaptation of spinal fracture treatments in patients with AS or DISH.

### 1. Fracture characteristics

AS or DISH usually accompany ankylosing spines. Noncontiguous spinal fractures in patients with ankylosing spine, as in the present case, are not rare [1, 5]. Because of poor bone quality, the spine is brittle, osteoporotic, and stiff [4, 6, 7], increasing susceptibility to fracture even with low-energy trauma [8, 9]. Fracture pattern in patients with AS typically involves three columns [10], as in this case. Compared with previous reports, this study is unique because the fracture occurred in an ankylosing spine with SED. The cervical spinal fracture in our patient was observed at the C6–C7 segmental level, consistent with previous reports [1, 4, 11]. Spinal fractures in patients with AS frequently accompany hyperextension factures [1, 11] and are classified into four patterns based on fracture excursion through intervertebral disc, vertebral body, or both [1]. However, coronal CT in our case demonstrated a gap between the left and right fracture lines at both cervical and thoracic vertebral bodies—a pattern not previously observed. Based on the accident and the difference of number of left and right rib fractures (4:1), we attribute the pattern of fracture lines to direct or indirect lateral external forces impacting the spine.

### 2. Etiology of ankylosing spine

Congenital SED is a rare form of skeletal systemic disease [12]. Previous studies have demonstrated that patients with congenital SED present with a variety of deformities in the spine, including instability of the atlanto-axial joint, progressive kyphoscoliosis, platyspondylitis, lordotic lumbar vertebrae, and pear-like shape to the corps vertebrae at the thoracolumbar interface [13, 14]. However, our patient had extremely rare features in the spine such as ankylosing spine and the absence of instability of the atlanto-axial joint with SED. To our knowledge, only two studies have reported ankylosing spines with SED [15, 16], wherein patients exhibited X-linked recessive inheritance and associated mutations of transport protein particle *TRPPC2* [17], with onset later than congenital SED. This patient probably had another causative gene because of her sex and appearance of spinal ankylosing and multiple joint contractures in infancy. Because SED is a skeletal disorder mediated by *COL2A1*, we investigated for gene mutations [18] but found none. Moreover, no mutation of *TRPV4*, reportedly related to spondylometaphyseal dysplasia (Kozlowski type) and SED (Maroteaux type) [19], was observed. The genetic etiopathology in our patient remains unknown.

### 3. General problems treating ankylosing spinal fractures

Ankylosing spine creates high instability at fracture site because of long lever arms secondary to spinal column stiffness. Fractures frequently occur at the C-T and T-L transitional zones, which are subjected to extension force when patients are supine, resulting in delayed union [20, 21], epidural hemorrhage, and/or late-onset paralysis [22, 23]. Furthermore, a much higher mortality rate is reported in patients with cervical spinal fracture who have AS (> 30%) [1, 11] than in those without AS (18%) [11]. Therefore, early aggressive surgeries with posterior and/or anterior fixation are recommended. A disadvantage of treating this fracture type is the increase in the number of fused spinal segments (average, 5.6 segments) [4]; it is generally recommended to make multiple anchor points by extending the instrumentation over at least three vertebral levels above and below the fracture site [4, 24].

### 4. Specific devices used for treatment

Our patient had extensive thoracic kyphosis; therefore, we carefully monitored motor- and somatosensory-evoked potentials intraoperatively. To prevent intraoperative worsening of spinal alignment in the prone position, secondary to contact pressure disparities at the frontal truncal surface due to the rigid and kyphotic spine, we inserted cushioning materials into the space between the trunk and operating table. A CT-based navigation system was used for placing pedicle screws at C2 and the thoracic spine, considering the extreme difficulty in identifying anatomical landmarks for pedicle screw insertion. We made C2 anchors because we were concerned that cervical lateral mass screws had significantly lower resistance to pull-out forces than pedicle screws [25]. No new fracture was observed in our case at the thoracic spine without instrumentation between T4 and T9 partly due to her lower activity level secondary to ankyloses of all joints and spine. However, even with similar fracture patterns, non-skip cervicolumbar fusion would be favorable in patients with higher activity levels. Eventually, bony fusion of three-column spinal fractures at C-T and T-L junctional zones using hybrid open and percutaneous spinal fusion techniques was successful, despite difficulties ensuing from SED and spinal ankyloses of idiopathic genetic etiopathogenesis.

## Abbreviations

SED: spondylo-epihyseal dysplasia
CT: computed tomography
AS: ankylosing spondylitis
DISH: diffuse idiopathic skeletal hyperostosis
C-T: cervico-thorcic
T-L: thoraco-lumbar
